# Asynchrony between Antarctic temperature and CO_2_ associated with obliquity over the past 720,000 years

**DOI:** 10.1038/s41467-018-03328-3

**Published:** 2018-03-06

**Authors:** Ryu Uemura, Hideaki Motoyama, Valérie Masson-Delmotte, Jean Jouzel, Kenji Kawamura, Kumiko Goto-Azuma, Shuji Fujita, Takayuki Kuramoto, Motohiro Hirabayashi, Takayuki Miyake, Hiroshi Ohno, Koji Fujita, Ayako Abe-Ouchi, Yoshinori Iizuka, Shinichiro Horikawa, Makoto Igarashi, Keisuke Suzuki, Toshitaka Suzuki, Yoshiyuki Fujii

**Affiliations:** 1 Department of Chemistry, Biology, and Marine Science, University of the Ryukyus, 1 Senbaru, Nishihara, Okinawa 903-0213 Japan; 2 National Institute of Polar Research, Research Organization of Information and Systems, 10-3 Midori-cho, Tachikawa, Tokyo 190-8518 Japan; 3 Department of Polar Science, The Graduate University for Advanced Studies (SOKENDAI), 10-3 Midori-cho, Tachikawa, Tokyo 190-8518 Japan; 4 Laboratoire des Sciences du Climat et l’Environnement (LSCE), Institut Pierre Simon Laplace, CEA-CNRS-UVSQ, Université Paris Saclay, UMR 8212 Gif-sur-Yvette, France; 5 Graduate School of Environmental Studies, Nagoya University, Nagoya, 464-8601 Japan; 6 Atmosphere and Ocean Research Institute, The University of Tokyo, Kashiwa, 277-8568 Japan; 7 Japan Agency for Marine-Earth Science and Technology, 3173-25 Showamachi, Kanazawa, Yokohama, Kanagawa 236-0001 Japan; 8 Institute of Low Temperature Science, Hokkaido University, Kita-ku, North 19 West 8, Sapporo, Hokkaido 0600819 Japan; 9 Nishina Center, RIKEN, Hirosawa 2-1, Wako, 351-0198 Japan; 10 Department of Environmental Sciences, Shinshu University, Asahi 3-1-1, Matsumoto, 390-8621 Japan; 11 Department of Earth and Environmental Sciences, Yamagata University, Kojirakawa 1-4-12, Yamagata, 990-8560 Japan; 12 Present Address: Research Department, Fukushima Prefectural Center for Environmental Creation, 10-2 Fukasaku, Miharu, Fukushima 963-7700 Japan; 13 Present Address: Department of Global Environmental Engineering, Kitami Institute of Technology, 165 Koen-cho, Kitami, Hokkaido 090-8507 Japan; 14 Present Address: Earthquake and Volcano Research Center, Graduate School of Environmental Studies, Nagoya University, Nagoya, Japan

## Abstract

The δD temperature proxy in Antarctic ice cores varies in parallel with CO_2_ through glacial cycles. However, these variables display a puzzling asynchrony. Well-dated records of Southern Ocean temperature will provide crucial information because the Southern Ocean is likely key in regulating CO_2_ variations. Here, we perform multiple isotopic analyses on an Antarctic ice core and estimate temperature variations at this site and in the oceanic moisture source over the past 720,000 years, which extend the longest records by 300,000 years. Antarctic temperature is affected by large variations in local insolation that are induced by obliquity. At the obliquity periodicity, the Antarctic and ocean temperatures lag annual mean insolation. Further, the magnitude of the phase lag is minimal during low eccentricity periods, suggesting that secular changes in the global carbon cycle and the ocean circulation modulate the phase relationship among temperatures, CO_2_ and insolation in the obliquity frequency band.

## Abstract (web summary)

The Antarctic temperature record displays a puzzling asynchrony with changes in CO_2_ through glacial cycles. Here, the authors show that a 720,000-year Antarctic temperature record is affected by variations in obliquity-induced local insolation that are associated with phase modulation of eccentricity cycle.

## Introduction

Precise knowledge of the relationship between changes in temperature, atmospheric CO_2_ and solar insolation is essential to understanding Earth’s climate system. The values of a temperature proxy, the hydrogen isotopic composition (δD), in the Antarctic EDC ice core^1,2^ have varied in parallel with CO_2_ concentrations over the past 800 thousand years (kyr; *r*^2^ = 0.82)^3^. However, δD apparently leads CO_2_ variations. For example, during the last termination (TI), the start of Antarctic warming has been estimated to be synchronous with CO_2_ increase^4^ or to lead CO_2_ increases by 800 ± 600 years^5^ on the East Antarctic Plateau. The lead is ca. 2000 years at a West Antarctic site^6^. Over the past 420 kyr, the Vostok ice core shows that the Antarctic δD temperatures lead the CO_2_ variations by 1.3 ± 1.0 kyr^7^. During the lukewarm interglacials (430–650 kyr BP), Antarctic δD leads CO_2_ by 1900 years, and the correlation between CO_2_ and δD is weaker (*r*^2^ = 0.57), as determined from the EDC core^8^.

Although the mechanisms underlying the coupling and the phase lags remain unclear, the Southern Ocean region, rather than Antarctica, is thought to play the central role in regulating CO_2_ variations^9,10^. A box model, for example, estimated a ca. 60% increase in CO_2_ during TI that is attributable to direct and indirect temperature effects, such as changes in sea ice cover and vertical mixing in the Southern Ocean^9^. On millennial time scales, a multi-proxy study suggests that an antiphased hemispheric temperature response to ocean circulation changes resulted in Antarctic temperatures leading global temperatures and CO_2_ during TI^11^. On orbital time scales, the latitudinal temperature differences between Antarctica and the surrounding ocean may arise from annual mean insolation (AMI) variations because the amplitude of AMI variations is largest in the polar regions and smallest in the mid-latitude regions^12–16^. The AMI is paced only by the 41-kyr periodicity of the Earth’s obliquity^17^. Indeed, a strong 41-kyr cycle that is likely related to the local AMI variations was recognized in the Antarctic temperature record obtained from the Vostok ice core in the 1980s^15^. However, uncertainties in the age models of the ice cores and ocean sediments limit our understanding of the potentially different time lags between Antarctic and Southern Ocean temperatures relative to AMI changes. Therefore, well-dated surface temperature records with high temporal resolution from the Southern Ocean provide crucial information.

In addition to these climatic mechanisms, the validity of δD as a temperature proxy should be considered. While this proxy depends on Antarctic site temperatures (Δ*T*_site_), it is also affected by the extent of fractionation-associated rainout from the water vapour as it is transported from its moisture source in the ocean to the site where the precipitation occurs. After correcting the δD variations for this moisture source effect, the Δ*T*_site_ record shows a stronger correlation with CO_2_ over the past 350 kyr^18^, suggesting a potential bias in the use of δD as a temperature proxy. However, the effects of moisture source corrections are unknown prior to 420 kyr BP^19^. The lack of long records prevents elucidation of the potential influence of the 400-kyr eccentricity cycle on the relationship among AMI, CO_2_ and Antarctic and ocean temperatures.

To investigate the relationship between temperatures and CO_2_ concentrations, we estimated Δ*T*_site_ and ocean surface temperatures (Δ*T*_source_) using new δD and deuterium excess records from the Antarctic Dome Fuji (DF) ice core, which spans the past 720 kyr. The new Δ*T*_site_ and Δ*T*_source_ records enable us to compare the pattern and timing of temperature variations between the Antarctic Plateau and its oceanic moisture source using the same age scale from that of the DF ice core. Our data extend the longest (420-kyr) record of Δ*T*_site_^19^ by 300 kyr, and they reveal the impact of moisture source corrections during the lukewarm interglacials. The correlation between Δ*T*_source_ and CO_2_ is stronger than that between δD and CO_2_. Over the entire periods covered by the extended records, changes in local solar insolation, paced by obliquity, strongly affects Δ*T*_site_. At the obliquity periodicity, the changes in Δ*T*_site_ and Δ*T*_source_ are delayed with respect to AMI. Further, the magnitude of the phase lag is minimal during the period of low orbital eccentricity, suggesting that the phase modulation is related to secular changes in the global carbon cycle and ocean circulations.

## Results

### Dome Fuji isotope and temperature records

**Fig. 1:**
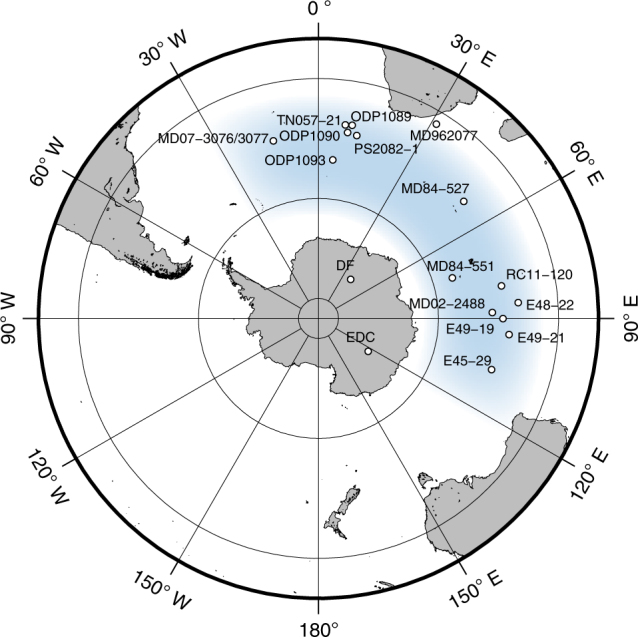
Locations of ice cores and marine sediment cores. Blue shading indicates the main present-day moisture source of the Dome Fuji site, as determined using back-trajectory calculations^24–26^. White dots indicate the locations of ocean sediments used to construct the stacked SST records. This map was generated using the Generic Mapping Tools^90^

We used the second DF ice core (DF2; 77˚ 19′ S, 39˚ 42′ E, 3810 m above sea level)^20^ to obtain a 720-kyr deuterium excess (*d* = δD–8δ^18^O) record. The δD and δ^18^O values of the ice between the depths of 2399.5 and 3035.1 m (297–715 kyr BP) were measured at 10-cm intervals. The new DF2 data were combined with earlier data from the first Dome Fuji core (DF1^21,22^) and were plotted on the AICC2012 time scale^23^. The relationships among δD, *d*, Δ*T*_site_ and Δ*T*_source_ were quantified using a mixed cloud isotopic model (Methods). In this study, temperatures are expressed as deviations, Δ, from the modern value (the average of the past 2 kyr). Δ*T*_source_ is a proxy for temperature in moisture source regions^19,22^, and the major moisture source for modern precipitation at DF is estimated to lie in the South Atlantic and Indian Oceans between 38 and 65˚ S, based on backward-trajectory calculations^24–26^ (Fig. 1).

**Fig. 2:**
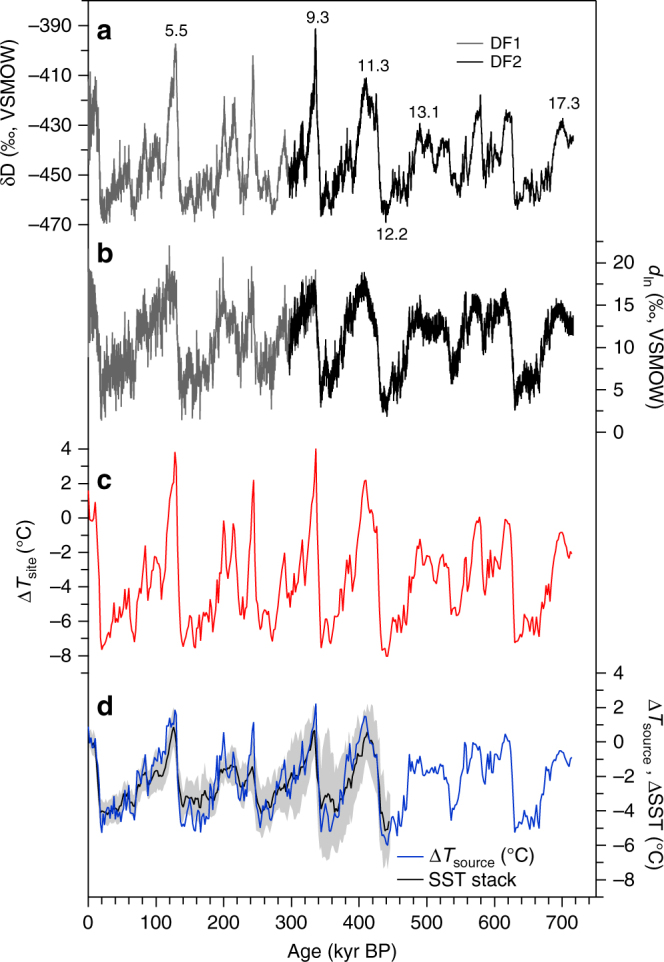
Isotope records from the DF1 and DF2 ice cores. Isotope records from DF1 (grey) and DF2 (black) for **a** δD and **b** *d*_ln_, as well as records of **c** Δ*T*_site_ (red) and **d** Δ*T*_source_ (blue) for DF. The black line indicates the stacked SST record within the moisture source region for precipitation (grey shading represents the range of variability) (Supplementary Fig. 4). Temperatures are shown as deviations from the modern value (i.e. the average of the past 2 kyr). The Δ*T*_site_ and Δ*T*_source_ records were resampled at a 2-kyr interval for clarity. Numbers indicate Marine Isotope Stages

The DF2 core provides detailed δD profiles over the past 720 kyr (Fig. 2). The δD record from DF covaries linearly with that from EDC^1,2^ (*R*^2^ = 0.91, with an EDC/DF slope of 1.1) over the past 720 kyr. With their higher sampling resolution (10 cm vs. 55 cm), the DF data depict the paleoclimate signals in greater detail than the EDC core. However, the deepest part appears to have been smoothed by ice diffusion, a process also observed in the 11-cm samples from the EDC core that correspond to Marine Isotope Stage (MIS) 11 (Supplementary Fig. 1).

The DF Δ*T*_site_ correlates well with DF δD at glacial–interglacial time scales over the entire record (Fig. 2). Millennial-scale events during glacial periods in the δD record are also identifiable in the Δ*T*_site_ record (Supplementary Fig. 2). The Δ*T*_site_ values range from −8.0 °C (MIS 12.2) to 4.0 °C (MIS 9.3) when 2-kyr mean values are used (Fig. 2). The Δ*T*_site_ record shows the less-pronounced interglacial maxima prior to ~430 kyr (the Mid-Brunhes Event, MBE), as suggested by the EDC δD variations^1,2^.

**Fig. 3:**
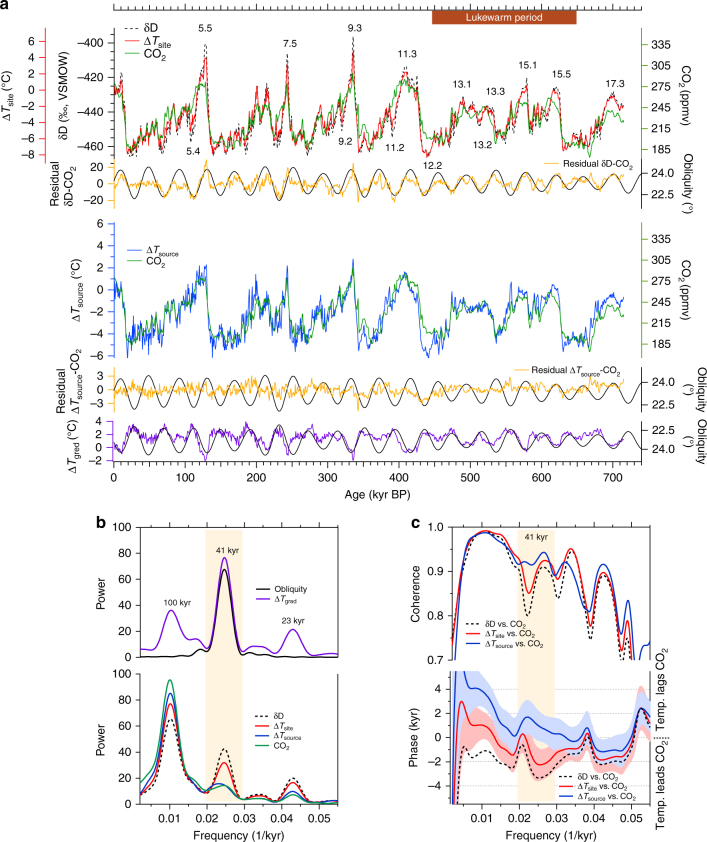
Temperature reconstructions from the DF2 core together with the CO_2_ record. **a** The upper panel shows the DF δD (black) and Δ*T*_site_ (red) records and the CO_2_ composite record^32^ (green), as well as the residuals from a linear regression between δD and CO_2_ (orange) and obliquity (black). The lower panel shows DF Δ*T*_source_ (blue) and the CO_2_ composite record (green). DF Δ*T*_grad_ (purple) with obliquity (black) is shown at the bottom. The δD (or Δ*T*_source_) axis was scaled to fit a linear regression between δD (or Δ*T*_source_) and CO_2_. Numbers indicate Marine Isotope Stages. **b** Power spectra of Earth’s obliquity (black) and DF Δ*T*_grad_ (purple), DF δD (black dotted), Δ*T*_site_ (red), Δ*T*_source_ (blue) and the CO_2_ composite record^32^ (green). **c** Upper panel: coherence of δD vs. CO_2_ (black dotted), Δ*T*_site_ vs. CO_2_ (red) and Δ*T*_source_ vs. CO_2_ (blue). Lower panel: same as upper panel but for phase lag. Red and blue colours indicate uncertainty ranges (Methods). The uncertainty range of CO_2_ vs. δD is similar to that of CO_2_ vs. Δ*T*_site_; however, it is not shown here for clarity. The obliquity band is shown in yellow

The DF2 core *d* record is shown in terms of the logarithmic definition of *d* (hereafter *d*_ln_; Fig. 2), which reflects moisture source conditions better than the traditional linear definition^22^ (Supplementary Fig. 3). Recent modelling studies also present evidence supporting the logarithmic definition^27,28^. The glacial–interglacial variations in Δ*T*_source_ are generally similar to the variations in *d*_ln_ (Fig. 2). The DF2 Δ*T*_source_ values range from −6.0 °C in MIS 12.2 to 2.2 °C in MIS 9.3. During glacial inceptions, Δ*T*_source_ remains warm, whereas Δ*T*_site_ begins to cool (for example, during MIS 11.2; Fig. 3a). Other features also emerge during the lukewarm interglacials. Notably, a ca. 2 °C drop in Δ*T*_site_ during MIS 13.2 has no counterpart in Δ*T*_source_ (Fig. 3a).

To estimate temperatures, we used the same parameters used in a previous study^22^ (Methods). The use of these values produces glacial–interglacial variations in Δ*T*_source_ that are larger than those presented by previous studies^19,29^, resulting in good agreement between the DF Δ*T*_source_ record and a stacked SST record^30^ over the past 150 kyr^22^. Here, to evaluate the reliability of our Δ*T*_source_ estimates over multiple glacial cycles, we constructed a stacked sea surface temperature (SST) record based on 17 individual deep-sea sediment cores obtained in the source region (Methods and Supplementary Fig. 4). The stacked SST record shows remarkable similarities with our Δ*T*_source_ record in terms of both amplitudes and the patterns of variation over the past 440 kyr, and they agree within mutual amplitude and age uncertainties (Fig. 2d). Hence, the effects of shifts in the moisture source associated with westerly wind displacement^28^ are limited in the DF record on glacial–interglacial time scales. Between the pre- and post-MBE periods, Δ*T*_source_ exhibits an increase in amplitude of only 17%, as estimated using standard deviations^1^. This result is consistent with the overall concept that changes associated with the MBE are less prominent at low latitudes than at high latitudes^31^. We thus interpret our Δ*T*_source_ reconstruction to be representative of the average changes in SSTs over broad regions of the oceanic moisture source.

## Discussion

Throughout the entire record, both Δ*T*_site_ and Δ*T*_source_ covary with CO_2_ (*R*^2^ = 0.78 and 0.77, respectively), and these correlations are significantly stronger than that between δD and CO_2_ (*R*^2^ = 0.70, see Methods). The only exception for the strong coupling between Δ*T*_source_ and CO_2_ is observed during MIS 12.2, during which the Δ*T*_source_ shows exceptional cooling (Fig. 3a). This observation suggests that Southern Ocean temperatures experienced exceptional cooling during MIS 12 (see discussion below).

The weakest correlation between δD and CO_2_ is observed in the EDC core during the lukewarm interglacials^8^. During this period, the correlation between DF Δ*T*_site_ and the composite CO_2_ record^32^ (*R*^2^ = 0.76) is stronger than that between δD and CO_2_ (*R*^2^ = 0.66, Fig. 3a). We also obtained a high correlation between the records of Δ*T*_source_ and CO_2_ concentrations during the lukewarm interglacials (*R*^2^ = 0.80, Fig. 3a). During MIS 13.2, for example, Δ*T*_source_ and CO_2_ remain at levels similar to those of MIS 13.1 and MIS 13.3, whereas Δ*T*_site_ exhibits a ca. 2 °C drop.

Despite the overall correlation between δD and Δ*T*_site_, the Δ*T*_site_ record lags the δD record by 0.9 ± 0.1 kyr in the obliquity band (Supplementary Fig. 5), which results in a smaller phase lag between Δ*T*_site_ and CO_2_ than that between δD and CO_2_ (Fig. 3c). Therefore, our data suggest that the lead in Antarctic δD temperatures (i.e. temperature without correcting for source effects) over CO_2_ is partly attributable to the effects of the moisture source on δD temperatures over the past 720 kyr in the obliquity band. These results suggest that the importance of moisture source effects for the obliquity signal in δD. Thus, the source effect must be considered in future research about the relationship between Antarctic temperatures and CO_2_.

The similarities and differences between temperatures and CO_2_ concentrations mainly emerge from the 41-kyr obliquity periodicity. Spectral analyses reveal a smaller power in the obliquity frequency range for CO_2_ and Δ*T*_source_ than for Δ*T*_site_ and δD (Fig. 3b). Indeed, periodic obliquity variations remain in the residuals from linear regression between δD and CO_2_, but not for Δ*T*_source_ and CO_2_ (Fig. 3a). Within this frequency range, the CO_2_ variations display higher coherence with Δ*T*_source_ than with Δ*T*_site_ or δD (Fig. 3c). These results show that, within the obliquity frequency band, the CO_2_ variations are not tightly coupled with temperatures on the East Antarctic Plateau (Δ*T*_site_ or δD). Rather, the correlation between CO_2_ and Δ*T*_source_ suggests a close link between surface temperature changes in the Southern Ocean and the global carbon cycle.

Despite the strong correlations between Δ*T*_source_ and CO_2_, Δ*T*_source_ lags CO_2_ slightly in most frequency bands (Fig. 3c), most likely because of the large heat inertia of the Southern Ocean^33^. Furthermore, DF Δ*T*_site_ leads Δ*T*_source_ by 2.8 ± 0.6 kyr (Supplementary Fig. 5) in the obliquity band. We should note that this analysis is focused on changes over orbital time scales, not millennial time scales, over which Antarctic warming is passively controlled by the Southern Ocean warming because of the thermal bipolar seesaw^33,34^. Within the obliquity frequency band, our analyses suggest that temperature variations in Antarctica have led ocean temperatures throughout the past 720 kyr. This phenomenon is most likely explained by the strong influence of local AMI on Δ*T*_site_.

A strong 41-kyr periodicity can be found in the temperature gradient record (Δ*T*_grad_ = Δ*T*_source_–Δ*T*_site_) (Fig. 3b). A decrease in obliquity increases the AMI gradient between the middle and high latitudes^17^, which covary in phase with the obliquity signal^17,35^. Thus, the changes in obliquity correlate closely with the AMI gradient between DF (77° S) and the moisture source region (38–66° S). The Δ*T*_grad_ record is negatively correlated with the obliquity (Fig. 3a). This result is qualitatively consistent with the latitudinal temperature pattern expected from the local insolation gradient.

**Fig. 4:**
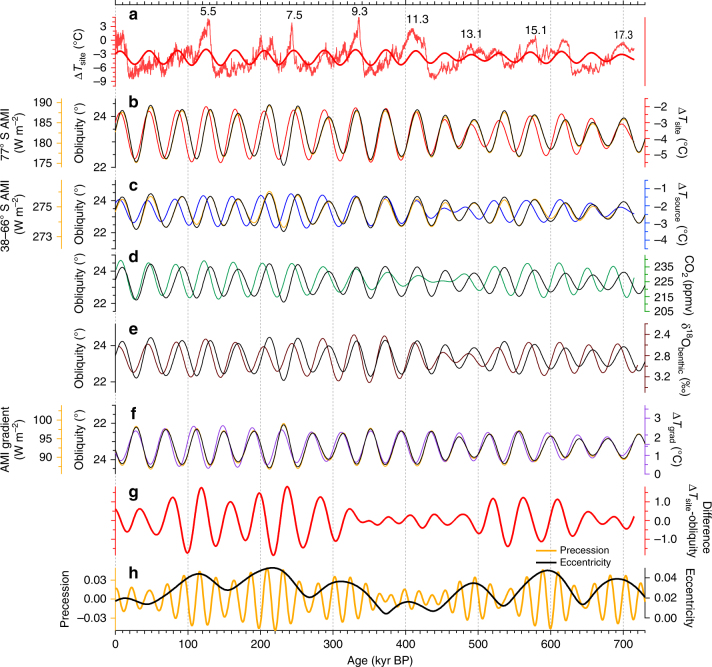
Obliquity components of climate records and orbital parameters. **a** DF Δ*T*_site_ (thin red) and its obliquity component (red). **b** The obliquity component of Δ*T*_site_ (red), AMI at 77° S (yellow) and obliquity (black). **c** The obliquity component of Δ*T*_source_ (blue), AMI at 38–66° S (yellow) and obliquity (black). **d** The obliquity component of the CO_2_ composite record^32^ (green) and obliquity (black). **e** The obliquity component of benthic δ^18^O record^40^ and obliquity (black). **f** The obliquity component of Δ*T*_grad_ (purple), the insolation gradient between 77° S and 38–66° S (yellow) and obliquity (black, axis inverted). **g** The differences between the normalized obliquity component of Δ*T*_site_ and AMI at 77° S (red). **h** Orbital eccentricity (black) and precession (yellow). The insolation curves are shown behind the obliquity variations. The obliquity components were calculated using a bandpass filter (Methods)

The impact of the differences in AMI on the amplitudes of Δ*T*_site_ and Δ*T*_source_ was evaluated by filtering the records in the obliquity band (Fig. 4). The range (i.e. difference between maximum and minimum) of the filtered Δ*T*_site_ record was 3 °C (Fig. 4b), which is close to the ca. 2.5 °C changes simulated by a general circulation model^13^. The amplitude of Δ*T*_site_ is twice as large as that of Δ*T*_source_ when it is filtered in the obliquity range (Fig. 4c). Although the large amplitude of Δ*T*_site_ expressed in the obliquity range is likely affected by AMI, the amplitude of AMI changes in the DF area is six times greater than that of the moisture source region (Fig. 4b, c). Accounting for the mean present-day planetary albedo, the effective shortwave forcing is reduced particularly strongly over Antarctica^12^. With this albedo effect, the site/source ratio for the surface net downward flux is 2.4:1 (Methods). Thus, the difference is significantly reduced, and roughly consistent with the Δ*T*_site_/Δ*T*_source_ ratio. Remaining difference would be balanced by other compensation mechanisms, such as the transport of heat from the low to the high latitudes^36^, and latitudinal contrasts in the cloud feedback, which is negative near 60° S and positive at low latitudes^37^.

The phase relations between the temperature proxies and AMI reveal a unique characteristic of the temperature responses to AMI. On average, Δ*T*_site_ is delayed with respect to AMI (or obliquity) by 4.1 ± 2.7 kyr at the obliquity period (Fig. 4b), as was previously shown for the EDC^2^ and DF1 cores^21^. The Δ*T*_source_ is also delayed relative to AMI by 7.5 ± 2.9 kyr at the obliquity period (Fig. 4c). Interestingly, the filtered Δ*T*_grad_ and the AMI gradient are almost synchronous (Fig. 4f). Their lag in the obliquity band is 1.9 ± 2.6 kyr, which lies within the uncertainty of the age scale^23^. This apparent synchronization can be simply explained by the fact that the delay in Δ*T*_site_ relative to AMI is cancelled out by that of Δ*T*_source_ relative to AMI. Thus, the Δ*T*_grad_ appears to be directly forced by the AMI gradient and obliquity without a significant phase lag. An increase in the AMI gradient enhances the degree of poleward moisture transport^35^, and results in increased snowfall and ice sheet volume in the Northern Hemisphere. Concurrently, an enhanced southward latent heat transport is expected to offset local, insolation-driven Antarctic cooling^12^. These positive and negative feedback mechanisms potentially result in the lead and lag of Antarctic temperatures relative to the AMI variations.

A closer look at the phase lag between Δ*T*_site_ and AMI reveals that the magnitude of the phase lag varies with the eccentricity period of ca. 400-kyr. Large delays in Δ*T*_site_ relative to AMI were observed during 100–300 kyr BP and 500–650 kyr BP (Fig. 4b). The phase lags become small when eccentricity is small (during 350–450 kyr BP), when Δ*T*_site_ and AMI are almost synchronous. To illustrate this 400-kyr phase modulation, the amplitudes of the filtered Δ*T*_site_ and AMI records were normalized to a standard deviation of 1, and the difference between them is shown in Fig. 4g. Because a smaller phase lag results in a smaller difference, the amplitude of the filtered Δ*T*_site_–AMI difference record reaches a minimum during 350–450 kyr BP (Fig. 4g).

As seen in the Δ*T*_site_ record, the obliquity-filtered Δ*T*_source_ and CO_2_ also show varying phase lags with respect to obliquity with a ca. 400-kyr eccentricity cycle (Fig. 4c, d). On average, the filtered CO_2_ record shows a delay of 7.0 ± 2.9 kyr with respect to obliquity. These phase lags are significant during most of the studied period because they are larger than the uncertainty of AICC2012, which ranges from ±1.7 to ±4.8 kyr (±2.6 kyr, on average) over the past 100–720 kyr^23^. Large uncertainties were expected during MIS 11-12 because of the lack of age markers^23^. However, an abrupt decrease in δ^18^O values seen in Chinese stalagmites dated independently using the U/Th technique^38^ coincides with an abrupt increase in EDC CH_4_^39^ at Termination V, which supports the accuracy of the AICC2012 age scale.

Interestingly, an independently dated benthic foraminiferal δ^18^O record also shows a similar varying phase with respect to obliquity^40^ (Fig. 4e). When filtered in the obliquity band, the benthic δ^18^O data suggest that the 41-kyr component of the ice volume changes in the Northern Hemisphere are also delayed relative to obliquity, and the ice volume and obliquity are almost synchronous at the period of the eccentricity minimum. Thus, the delayed responses relative to obliquity are observed not only for Δ*T*_site_, but also for Δ*T*_source_, CO_2_ and a proxy for ice volume. Therefore, our data do not support the hypothesis of a direct influence of local solar insolation on Antarctic temperatures^15,16^ without any link to the Northern Hemisphere^16^. Rather, the millennial-scale time delay in Δ*T*_site_ relative to AMI suggests the influence of ‘slow’ feedbacks, such as changes in greenhouse gases and/or global ice area/volume^41,42^ during periods of large eccentricity.

We next attempt to determine whether ice volume or CO_2_ is more directly related to the varying phase lag at the 400-kyr periodicity. Although modelling results suggest that the Eurasian ice sheet responds to insolation forcing at the 41-kyr and 23-kyr periodicities without a significant 100 kyr cycle^41^ and that Antarctic ice volume varied with a periodicity of 400-kyr^43^, little evidence indicates a link between the phase lag and AMI. In an experiment with constant CO_2_, an ice sheet model could not reproduce the decrease in ice volume during MIS 11 because of the small amplitude of precession^44^. This result suggests that changes in ice volume are sensitive to atmospheric CO_2_ changes when eccentricity is low. Therefore, in spite of the many uncertainties, we suggest that changes in CO_2_, rather than that of ice volume, are the primary driver of the phase modulation with the 400-kyr periodicity.

The phase modulation of CO_2_ over a 400-kyr-long cycle is linked to the reduced amplitude of precession variations during periods of low eccentricity (Fig. 4h), which result in a relatively strong obliquity effect. During TI, CO_2_ rose at ~18 kyr BP, which is related to the melting of the Northern Hemisphere ice sheet and the subsequent weakening of the Atlantic meridional overturning circulation (AMOC)^11^. Thus, the timing at which CO_2_ begins to rise during a termination would be determined by when the Northern Hemisphere ice sheet begins to melt. When eccentricity is small, the summer insolation maxima are small. Thus, if obliquity rises beyond the threshold of melting, a moderate climate forcing could cause warming enough that the southern margin of the North American ice sheet begin to retreat^41^. Therefore, from 350 to 450 kyr BP, the obliquity component of CO_2_ began to rise rapidly without a phase difference relative to obliquity.

**Fig. 5:**
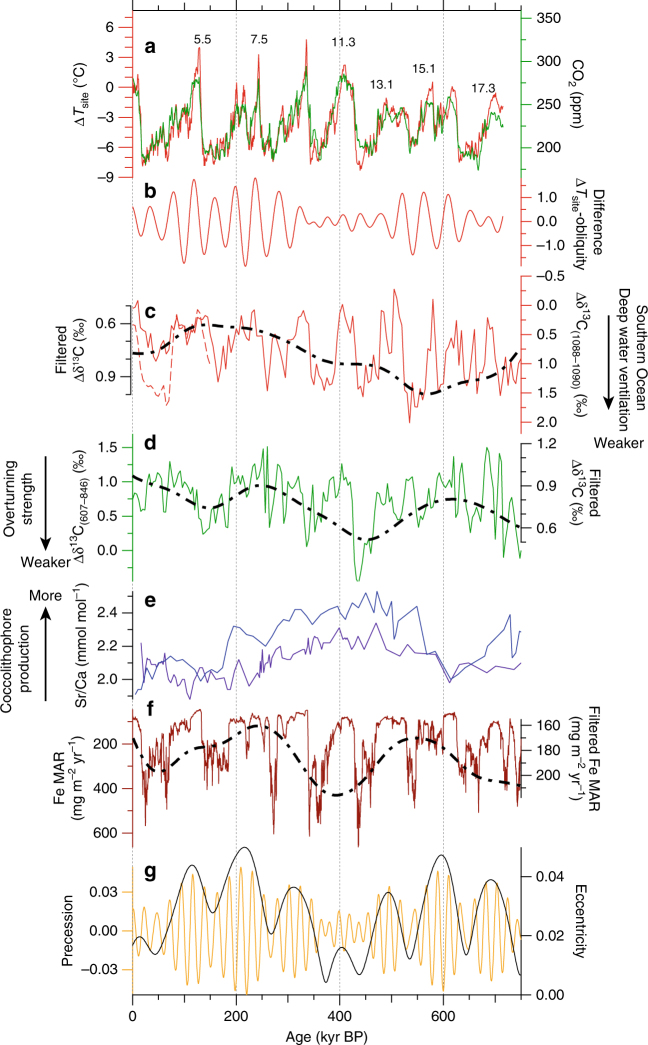
Link between the 400-kyr phase modulation and the global ocean carbon cycle. **a** DF Δ*T*_site_ (red) and the composite atmospheric CO_2_ record^32^ (green). **b** The difference between the normalized obliquity component of Δ*T*_site_ and AMI (same as in Fig. 4g). **c** The intermediate-to-deep δ^13^C gradient of benthic foraminifera (Δδ^13^C), a proxy for deep-water ventilation in the Southern Ocean^49^. Δδ^13^C represents the difference in δ^13^C between two cores, specifically those collected at ODP site 1088 (ca. 2100 m water depth) and site 1090 (ca. 3702 m water depth) in the South Atlantic Ocean. Dotted red line indicates the Δδ^13^C obtained using *C. kullenbergi* data at site 1090 and solid red line indicates *C. wuellerstorfi* data only. **d** A measure of overturning strength based on δ^13^C gradient^50^ between the North Atlantic (ODP site 607)^91^ and the tropical Pacific Ocean (ODP site 846)^92^. Small gradients (close to zero) imply drastically reduced ventilation of the Atlantic or enhanced ventilation in the Pacific^50^. **e** Sr/Ca of coccolithophores as a proxy for the rate of coccolithophore production in southwestern Indian Ocean (purple line, MD962077) and in the western equatorial Pacific (blue line, V28-239)^45^. **f** Mass accumulation rate (MAR) of iron in the subantarctic Atlantic Ocean (ODP site 1090)^48^. Dashed-dotted black line indicates the long-term trend obtained using a Gaussian filter around 0 kyr^−1^ with a 0.0045 kyr^−1^ bandwidth (i.e with periodicity < 222 kyr removed). **g** Orbital eccentricity (black) and precession (yellow)

Besides the timing of deglaciation, the ca. 400-kyr long cycle of the global ocean carbon cycle may also modulate the phase relationship. A pronounced increase in coccolith production, which cause atmospheric CO_2_ to increase, occurs at the time of low eccentricity^45^ (Fig. 5e). Paradoxically, the amplitudes of changes in the atmospheric CO_2_ records from ice cores remain relatively stable at that time (Fig. 5a), suggesting that the enhanced efflux of CO_2_ may have been counterbalanced by high total marine productivity^46,47^, which would be linked with enhanced iron fertilization during MIS 8, 10 and 12 in the South Atlantic Ocean^48^ (Fig. 5f). These data imply an irregular enhancement CO_2_ exchange between the atmosphere and the ocean from 350 and 450 kyr BP, which would have contributed to the small amplitude of CO_2_ variations in the obliquity band between 350 and 450 kyr BP.

We suggest that a reduction in the response time of the ocean to changes in solar insolation during the periods of low eccentricity may have produced the reduction in the phase difference between insolation forcing, Δ*T*_site_ and Δ*T*_source_ in the obliquity band. It has been suggested that, during glacial periods, carbon becomes sequestered in the deep Southern Ocean through reduced deep-water ventilation and enhanced nutrient utilization^10^. In fact, the intermediate-to-deep δ^13^C gradient in benthic foraminifera (Δδ^13^C), which is a proxy for deep-water ventilation in the South Atlantic Ocean, shows weaker deep-water ventilation during glacial times with a clear 100-kyr glacial cycle;^49^ this gradient also displays a secular 400–500-kyr-long trend in the glacial minima from MIS 6 to MIS 14 (Fig. 5c). Further, the record of Δδ^13^C between the North Atlantic and the tropical Pacific Ocean, which is a measure of overturning strength, shows a similar secular trend, and the lowest Δδ^13^C value occurs during MIS 12 (Fig. 5d). These results imply a drastically reduced ventilation of the Atlantic or enhanced ventilation in the Pacific^50^. The above studies suggest that the deep ocean water was isolated from the surface-intermediate ocean during these periods because of weakened ocean circulation. The separation from the large heat reservoir of the deep oceans would have resulted in relatively fast thermal exchange between lower atmosphere and the surface ocean and a more rapid response of surface water temperatures to changes in insolation.

The reduced ventilation of the Atlantic (Fig. 5d) is linked with the exceptional cooling seen in Δ*T*_source_ during MIS 12.2 (Fig. 3a). This exceptional cooling of the Southern Ocean temperatures was likely associated with anomalously cool tropical SSTs that occurred at that time and were caused by northward movement of the subtropical front (STF)^50^. The annual maximum and minimum of daily equatorial insolation has pronounced eccentricity periodicities^51^. The low maximum equatorial insolation, during the modern period and 400 kyr ago, could reduce the strength of the Hadley cell, resulting in northward migration of the STF; this process likely also triggered the maximal reduction in North Atlantic deep-water formation during MIS 12 and MIS 10^50^.

Our temperature data reveal that the asynchrony between Antarctic temperature and CO_2_ has been associated with obliquity over the past 720,000 years. Large obliquity-induced variations in AMI strongly affect Antarctic temperature. Moreover, this obliquity effect is modulated by the eccentricity periodicity. During periods of low eccentricity, the delayed responses of Δ*T*_site_, Δ*T*_source_ and CO_2_ relative to AMI are minimal in the obliquity band. One possible explanation for this phenomenon is that the secular changes in the global ocean carbon cycle affect changes in ice volume, particularly during eccentricity minima. The secular changes in ocean circulation result in the isolation of the large heat reservoir of the deep ocean, and contribute to the reduction in the phase difference between AMI, Δ*T*_site_ and Δ*T*_source_ in the obliquity band. The Earth’s climate system is presently in a period of reduced eccentricity like the one that occurred at ~400 kyr BP. In the absence of human activities, the decrease in obliquity (and thus AMI at high latitudes) during the next 10 kyr would cause temperatures on the East Antarctic Plateau to decrease by 1–2 °C in the obliquity frequency band. The obliquity-paced solar radiation is also important as a driver of changes in glacier mass balance in Antarctica^52^. Thus, it is crucial to understand the climate mechanisms that underlie the apparent 400-kyr phase modulation of AMI relative to temperatures, CO_2_ and ice volume.

## Methods

### Samples and isotope measurements

The DF2 ice core was drilled to near bedrock at a depth of 3035.2 m^53^ at the DF station in East Antarctica. The drilling site is located at a summit of the East Antarctic ice sheet and has a current annual mean air temperature of −55 °C. Extensive analyses of the deepest part of the ice (3000.0–3035.0 m) indicate that the bottom ice originated from meteoric water with altered chemical compositions^54^.

Measurements of δD and δ^18^O values were performed for 6349 samples, which were obtained at depths ranging from 2399.5 to 3035.0 m. Each sample was 10 cm in length, which corresponds to a mean temporal resolution of ~70 yr. To remove surface contamination, 13 wt% of the surface was removed from each 24-g ice sample with a ceramic knife. The ice samples were melted at room temperature in sealed polyethylene bags. The water was then transferred to glass bottles and preserved in a frozen state. Both δD and δ^18^O values were measured using an automated equilibration method^55^ at the National Institute of Polar Research, Japan. The δ^18^O values of the ice samples (δ^18^O_ice_) were used in this study, whereas the δ^18^O values of ‘sawdust’ samples (δ^18^O_saw_) were used in the previous DF article^20^. The sawdust was collected during ice cutting at the DF site. The average difference (=δ^18^O_saw_–δ^18^O_ice_) and its variations (±1*σ*) were 0.31 ± 0.18‰ (*n* = 15), respectively. The small enrichment in δ^18^O_saw_ was caused by sublimation during sampling and/or transport of the samples from Antarctica to Japan. In addition, the resolution of this study is five times higher than that of the 50-cm sampling used in the previous DF2 δ^18^O_saw_ data^20^.

The isotope data are given using conventional *δ* notation: *δ* = *R*_sample_/*R*_VSMOW_−1, where *R*_sample_ and *R*_VSMOW_ are the isotopic ratios (D/H and ^18^O/^16^O) of the sample and Vienna Standard Mean Ocean Water (VSMOW), respectively. To improve the precision of the measurements, 45% of the samples were measured two to four times. The analytical precisions (1*σ*), which are based on the differences between the duplicate analyses of individual samples, were 0.44‰ (δD), 0.05‰ (δ^18^O) and 0.52‰ (*d*).

### Combining the record and the time scale

To obtain a complete isotopic record over 720 kyr, we used published data sets from the first Dome Fuji (DF1) core for shallow depths ranging from 0.0 to 2399.5 m. For the portion of the record derived from the DF1 core^21,56–58^, the resolution was ca. 400 years, and some parts were discontinuous. The overlapping portion (2400–2500 m) of the DF1 and DF2 records exhibited remarkable similarity^22,59^ because the horizontal distance between the two sites was only 43 m. The combined record was obtained by replacing the DF1 data^56–58^ with the DF2 data within the depth interval where the two cores overlap^22^ and adding the DF2 record. In this study, the DF records were plotted on the AICC2012 age scale^23,60^. To transfer the DF data to the AICC2012 age scale^23^, volcanic tie points between DF and EDC were used for 0–216 kyr BP^61^. For 216–720 kyr BP, the isotopic profiles were synchronized using the Match software package^62^. The glacial inceptions were excluded from the predetermined tie points^20^ because of potential differences in the patterns of the two isotopic profiles^63^. The largest mismatch between DF2 and EDC was found during 600–610 kyr BP. Isotope matching was impossible for this part of the record because of substantial differences in the observed trends.

### Temperature reconstruction

Traditionally, δD is used as a proxy for surface air temperatures, based on the spatial linear correlation between δD measurements and Antarctic temperatures^64^. However, the δD and δ^18^O variations in Antarctic ice cores^1,2^ depend on the extent of fractionation-associated rainout from atmospheric water vapour along the pathway from the oceanic moisture source to the precipitation site, as well as the site temperature (Δ*T*_site_). To estimate the site air temperature (Δ*T*_site_), including a correction for the temperature of the moisture source regions (Δ*T*_source_), a linear inversion method was used. This method has previously been applied to the Vostok core^18,29^, the EDC core^65^ and the DF core^22^. The sensitivities of the δD and *d* values to DF site air temperatures (Δ*T*_site_) and the temperatures within the moisture source region (Δ*T*_source_) were estimated using a mixed cloud isotopic model^66^. The multiple linear regression is expressed using the following equations:

1 $${\Delta \delta} {{\mathrm{D}}_{\mathrm{corr}}} = {{\gamma }}_{{\mathrm{site}}}{{\Delta T}}_{{\mathrm{site}}} - {\mathrm{\gamma }}_{{\mathrm{source}}}{{\Delta T}}_{{\mathrm{source}}}$$

2 $${{\mathrm{\Delta} d}}_{\rm{corr}} = - \beta _{{\mathrm{site}}}\Delta {{T}}_{{\mathrm{site}}} + \beta _{{\mathrm{source}}}\Delta {{T}}_{{\mathrm{source}}}$$

where Δ represents the deviation from the modern (where the modern is defined as the average value for the past 2 kyr), and the subscript ‘corr’ indicates the ice core record corrected for the isotopic composition of ocean water (Δ^18^O_sw_)^67^ using the Rayleigh equation^68^. For the temperature sensitivity parameters, *γ*_site_, *γ*_source_, *β*_site_ and *β*_source_, we used the values given by Uemura et al.^22^. Based on Monte Carlo simulations performed using the Vostok core, the uncertainties in the glacial–interglacial magnitudes of Δ*T*_site_ and Δ*T*_source_ are 1.1 °C and 0.8 °C, respectively^29^. The uncertainties for the DF site should be similar to those at Vostok because both are located in the inland portion of East Antarctica.

The above equations were written with a traditional linear definition of *d* to allow direct comparison with previous publications. Note that the definition of the *d* value does not affect the reconstructions for Δ*T*_site_ and Δ*T*_source_ values. Instead, it affects only the sensitivity parameters^22^. A comparison of the Δ*T*_source_, *d* and *d*_ln_ shows that Δ*T*_source_ resembles the variations in *d*_ln_ more closely than those of *d* for glacial–interglacial cycles (Supplementary Fig. 3).

In addition to Δ*T*_source_, relative humidity may affect the *d* values. In fact, present-day observations of water vapour in the Southern Ocean show that *d* depends on both SSTs and relative humidity^69^. Based on a Rayleigh-type model, the dependency of *d* on relative humidity has been estimated to be −0.15‰/% at Vostok^19^ and −0.045 to −0.095‰/% in East Antarctica^70^. An atmospheric general circulation model does not produce significant changes in relative humidity between the present day and the last glacial period^71^. A state-of-the-art climate model that includes coupled atmosphere, ocean and land processes also revealed changes in relative humidity of only ±5% over the ocean surface in the Southern Hemisphere^72^. Based on this relative humidity estimate and the sensitivity values, the changes in relative humidity lead to changes in *d* of ±0.23 to ±0.75‰, which is not significant when compared with the ca. 6‰ amplitude of the glacial cycles (Supplementary Fig. 3). Furthermore, general circulation models generally support the interpretation of Δ*T*_source_ in Antarctica^73,74^.

### SST records

In total, 17 SST records from ocean sediments^50,75–86^ within the DF moisture source region (Fig. 1) were collected from published databases. Various proxies (alkenones, Mg/Ca and faunal) were used to reconstruct the SSTs within that region. Six of the profiles reflect annual mean SSTs, whereas nine of the profiles reflect summer SSTs. Two of the profiles were produced by averaging summer and winter SST records^85^. The SST values are expressed in terms of deviations from the modern value, ΔSST (Supplementary Fig. 4), as with Δ*T*_source_. In this study, the average values for the past 2–5 kyr were used as the modern values. For MD07-3077, which does not contain data from the Holocene, modern SST data from the same site, MD07-3076, were used. In the case of MD02-2488, for which the SST data begin at 49 kyr BP, the present-day summer SST value was used as the modern value. Each ΔSST data series was resampled at a 2-kyr interval with linear interpolation. A stacked ΔSST record was then obtained by averaging the data without tuning the original age scales. The uncertainty in ΔSST was estimated by multiplying the standard error (*σ*/√*n*) by the *t*-value (97.5% confidence level). The stacked SST profile and uncertainty intervals are shown only for the period during which multiple data records (4 or more) were available (Fig. 1 and Supplementary Fig. 4).

### Albedo data

The albedo climatology was derived from data collected by the TIROS-N satellite. This data set was obtained from the climate data archive of the Joint Institute for the Study of the Atmosphere and Ocean (http://www.jisao.washington.edu/). The albedos of the DF region and the moisture source region are 0.78 and 0.46, respectively.

### Significance of the correlation coefficient

The uncertainty of the linear correlation coefficient was estimated using the Fisher *Z*-transform. For the entire record, the 90% confidence ranges of the correlations (*R*^2^) of CO_2_ with δD, Δ*T*_site_ and Δ*T*_source_ are 0.68–0.73, 0.76–0.80 and 0.75–0.79, respectively. For the lukewarm period, the ranges of the correaltion of CO_2_ with δD, Δ*T*_site_ and Δ*T*_source_ are 0.60–0.70, 0.72–0.79 and 0.77–0.83, respectively. Thus, the correlation between Δ*T*_site_ (and Δ*T*_source_) and CO_2_ is significantly stronger than that between δD and CO_2_. The correlation between Δ*T*_source_ and CO_2_ is slightly higher than that between Δ*T*_site_ and CO_2_, but it is statistically insignificant.

### Spectral analyses and the obliquity component

Spectral analyses were conducted with the Blackman–Tukey method (30% lag) using the Analyseries software package^87^ (Fig. 3 and Supplementary Fig. 5). The amplitudes of the records were normalized to a standard deviation of 1. The data shown in Fig. 3 were resampled using linear interpolation to a 700-yr interval, which is the average resolution of the composite CO_2_ record, and then smoothed using a 1-kyr cutoff low-pass filter. The data in Fig. S5 were resampled using linear interpolation to a 100-yr interval. The insolation data^88^ and the 41-kyr obliquity components (Fig. 4) were calculated using a Gaussian filter using the Analyseries software package (*f *= 0.0244 (1/kyr) with a bandwidth of 0.00488).

The uncertainties associated with the phase analysis were of three types: (i) ice-age versus ice-age phase analysis error (*σ*_phs_). The relative lags between the temperature proxies were as reliable as possible because they were estimated from the same ice sample. The phase analysis between Δ*T*_site_ and Δ*T*_source_ (Supplementary Fig. 5) is shown with a 90% confidence interval. (ii) Ice-age versus gas-age phase analysis error (*σ*_CO2_). Determining the exact phase lag between CO_2_ concentrations and temperatures was complex because the air was younger than the surrounding ice. A phase analysis between the temperature proxies and CO_2_ (Fig. 3) is shown with a 90% confidence interval (i.e. the error described in (i)) and an additional uncertainty associated with the gas-age/ice-age difference of ±0.55 kyr. This additional uncertainty was estimated using a gas-age/ice-age difference of 1.9–5.5 kyr^8^ and a 10% uncertainty in the age difference^89^, which approximately doubled the analytical uncertainty of the phase between Δ*T*_site_ and Δ*T*_source_. (iii) Ice-age versus orbital-age phase analysis error (*σ*_orb_). To compare with the variation in obliquity, the absolute age uncertainty of AICC2012 for ice must be considered. The uncertainty of AICC2012 is ±2.6 kyr on average during the past 100–720 kyr^23^. The phase lag and confidence intervals (90%) of Δ*T*_site_, Δ*T*_source_ and Δ*T*_grad_ with respect to obliquity were 4.1 ± 0.7, 7.5 ± 1.3 and 1.9 ± 0.3 kyr, respectively. For example, a combined error estimate for Δ*T*_site_ that is based on the root mean squared error is ±2.7 kyr. Therefore, the 4.1-kyr lag of Δ*T*_site_ against obliquity was significant during most of the studied period. The error in the gas-age versus orbital-age phase analysis is similar to *σ*_orb_ because the uncertainty of the AICC2012 age scale for gas is ±2.6 kyr, on average, during the past 100–720 kyr^23^.

### Data availability

The raw data used in this study are available at the NOAA’s National Centers for Environmental Information (NCEI), which is formerly the National Climate Data Center (NCDC) data archive (https://www.ncdc.noaa.gov/paleo/study/23371). The data are also available from the authors.

## Electronic supplementary material

Supplementary Information
