# Supplementary material for: Asynchrony between Antarctic temperature and CO2 associated with obliquity over the past 720,000 years
Source: Nat Commun. 2018 Mar 6;9:961. doi: 10.1038/s41467-018-03328-3 (PMC5840396; doi:10.1038/s41467-018-03328-3)
Supplement: Supplementary file 1 — Supplementary Information [file 41467_2018_3328_MOESM1_ESM.pdf]

Supplementary Information

Asynchrony between Antarctic temperature and CO_2 associated with obliquity over the past 720,000 years

Uemura et al.

Supplementary Figures 1-5
References for Supplementary Information

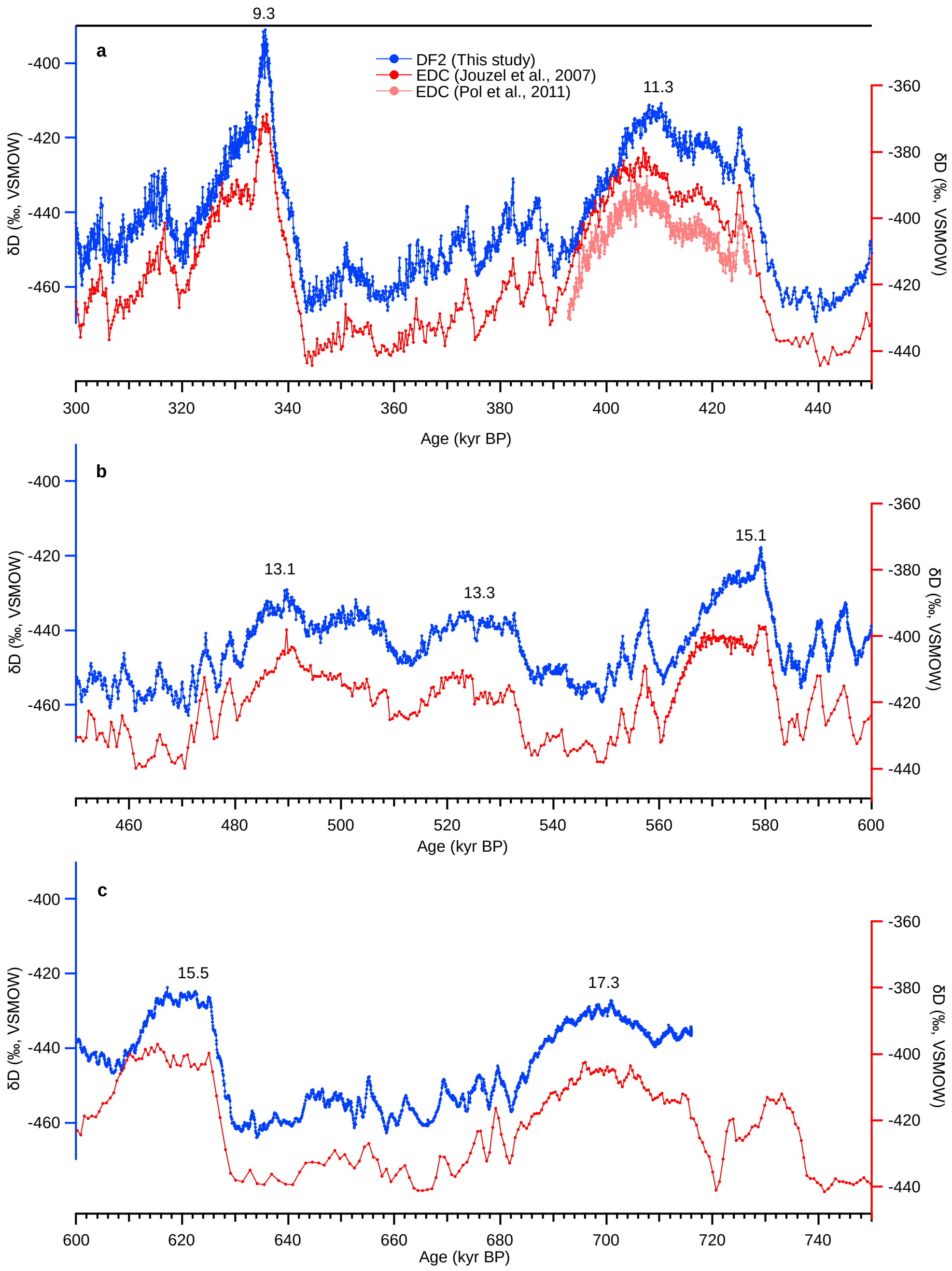

Supplementary Figure 1. δD records from the DF2 and EDC ice cores

The δD records of the DF2 (10-cm sampling, blue) and EDC cores (55-cm sampling, red)[1] for (**a**) 300-450 kyr BP, (**b**) 450-600 kyr BP and (**c**) 600-750 kyr BP. The light-red line indicates high-resolution (11-cm sampling) data[2] covering MIS 11 (these δD values are shifted by 10‰ for clarity). Numbers indicate Marine Isotope Stages. Dots represent raw data.

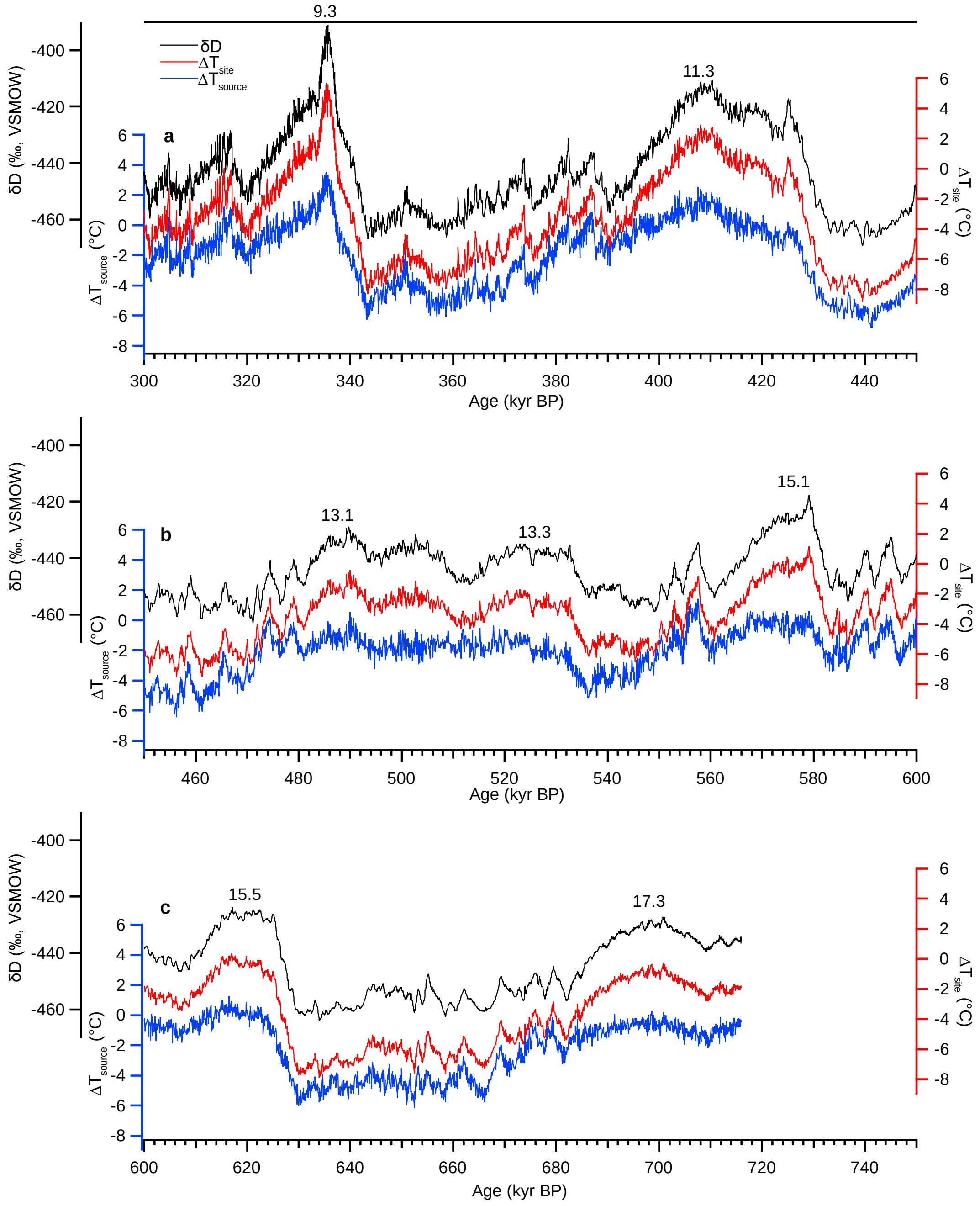

Supplementary Figure 2. Enlarged profiles of the δD, ΔT_{site} and ΔT_{source} records from the DF2 core

Records of δD (black), ΔT_{site} (red) and ΔT_{source} (blue) from the DF2 core for (**a**) 300-450 kyr BP, (**b**) 450-600 kyr BP and (**c**) 600-750 kyr BP. Numbers indicate Marine Isotope Stages.

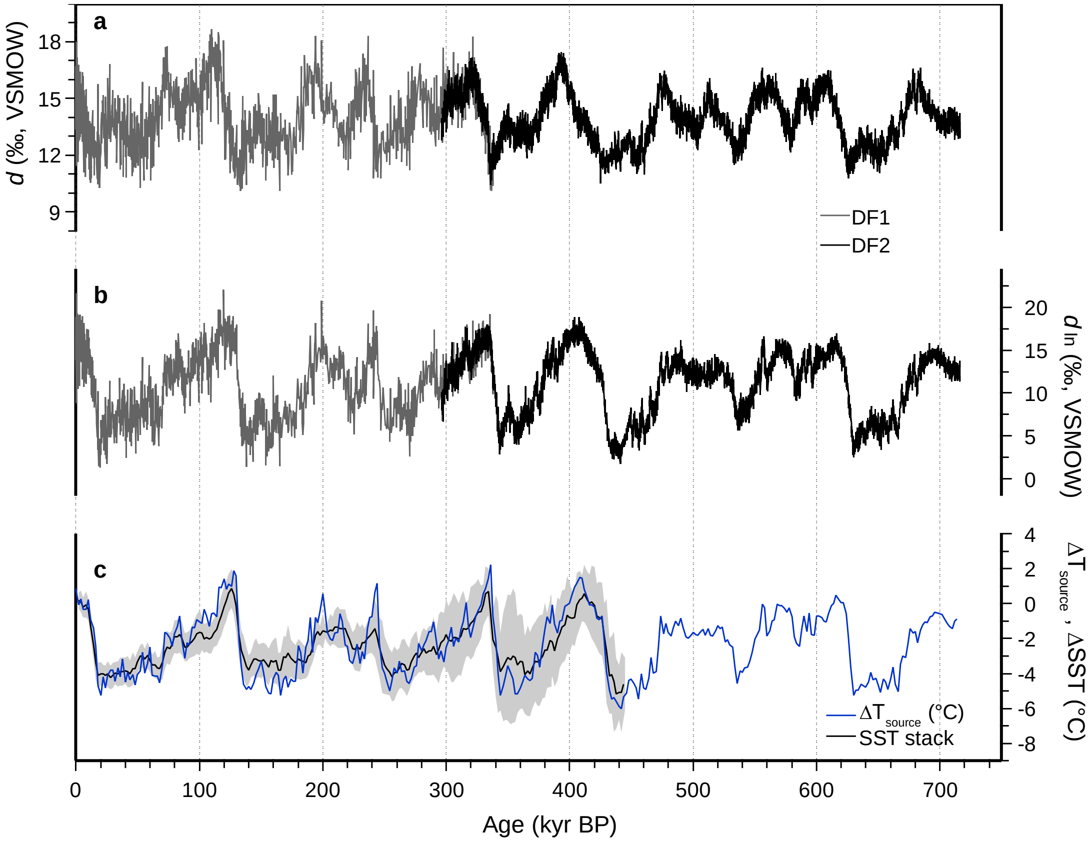

Supplementary Figure 3. Deuterium excess and ΔT_{source} records from the DF1 and DF2 ice cores

(**a**) The *d* record using the traditional linear definition[3] of the DF1 (grey) and DF2 (black). (**b**) Same as (**a**), but for the logarithmic definition of *d*, d_{ln}. (**c**) DF ΔT_{source} record (blue) shown with the stacked SST record (black) with its uncertainty interval (area shaded grey).

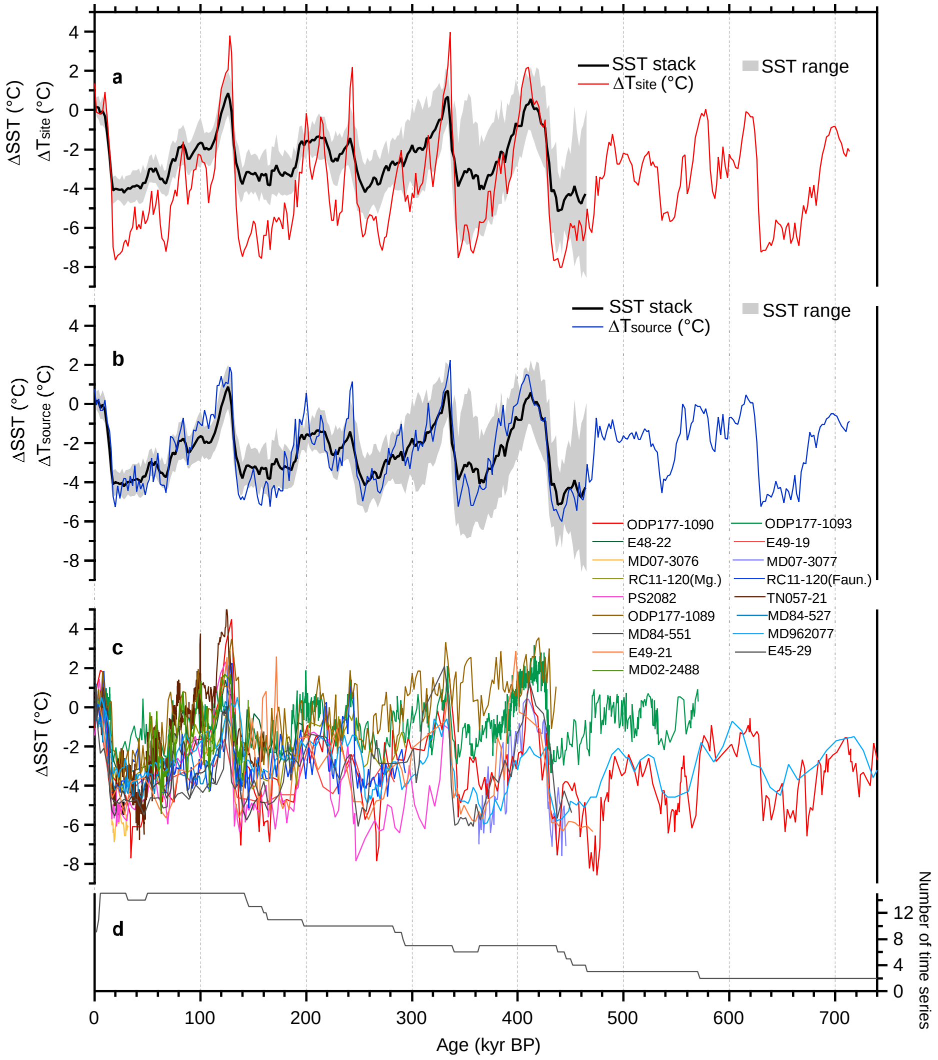

Supplementary Figure 4. DF ΔT_{source} and SST records from the moisture source region

(**a**) ΔT_{site} (red) record from the DF cores. The black line indicates the stacked SST record from the moisture source region with its uncertainty interval (area shaded grey). Data were resampled at a 2-kyr interval. (**b**) Same as (**a**), but for the ΔT_{source} (blue) record from the DF cores. (**c**) Individual SST records used to produce the stacked SST record (Methods). (**d**) Number of SST data series.

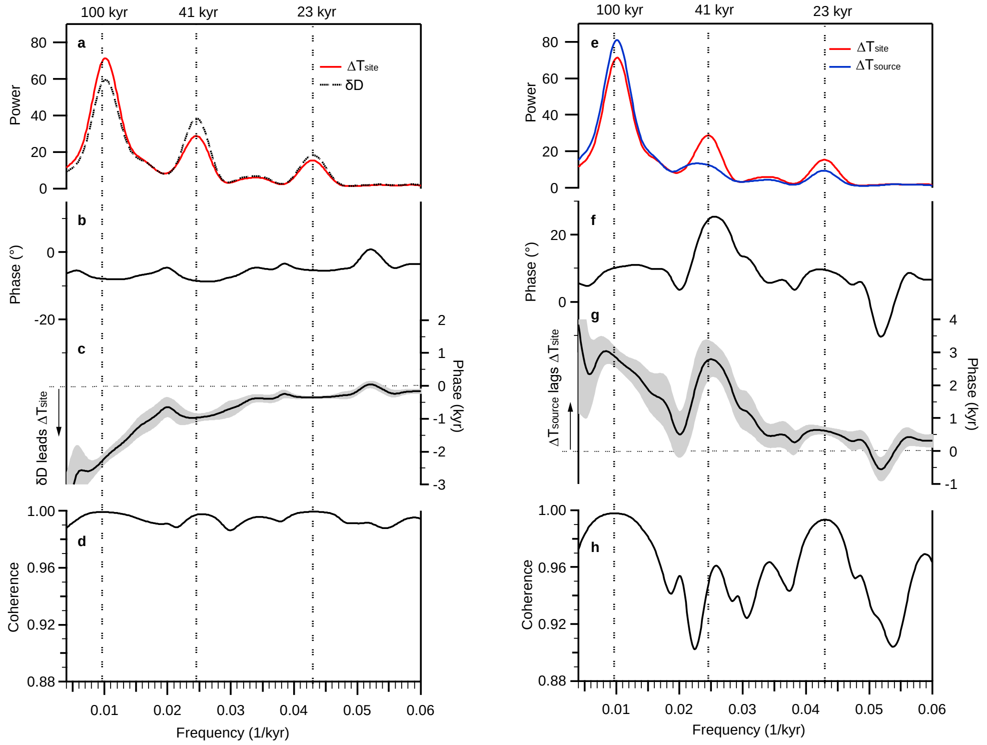

Supplementary Figure 5. Cross-spectral analyses of ΔT_{site} vs. δD and ΔT_{site} vs. ΔT_{source}

(**a**) Power spectrum of ΔT_{site} (red) and δD (black dotted). (**b**) Phase shown in units of degrees. (**c**) Same as (**a**) but shown using units of kyr. Negative values indicate that δD leads ΔT_{site}. (**d**) Coherence between ΔT_{site} and δD. (**e**)-(**h**), same as (**a**)-(**d**) but for ΔT_{site} (red) and ΔT_{source} (blue). Positive values indicate that ΔT_{source} lags ΔT_{site}. The spectral analyses were conducted using 100-yr resampled data.

Supplementary References

1. Jouzel, J. *et al.* Orbital and Millennial Antarctic Climate Variability over the Past 800,000 Years. *Science* **317**, 793-796, doi:10.1126/science.1141038 (2007).
2. Pol, K. et al. Links between MIS 11 millennial to sub-millennial climate variability and long term trends as revealed by new high resolution EPICA Dome C deuterium data - A comparison with the Holocene. *Climate of the Past*, **7**, 437-450, doi:10.5194/cp-7-437-2011 (2011).
3. Dansgaard, W. Stable isotopes in precipitation. *Tellus*, **16**, 436-468 (1964).
